# Supplementary material for: Beeswax waste improves the mycelial growth, fruiting body yield, and quality of oyster mushrooms (Pleurotus ostreatus)
Source: PeerJ. 2024 Dec 16;12:e18726. doi: 10.7717/peerj.18726 (PMC11657188; doi:10.7717/peerj.18726)
Supplement: Supplemental Information 1 [file peerj-12-18726-s001.docx]

| Treatment | CK (mm d^−1^) | T1(mm d^−1^) | T2(mm d^−1^) | T3(mm d^−1^) | T4(mm d^−1^) |
| --- | --- | --- | --- | --- | --- |
| 1 | 5.96 | 6.77 | 7.05 | 6.63 | 6.12 |
| 2 | 6.13 | 6.68 | 7.13 | 7.21 | 6.76 |
| 3 | 5.95 | 7.05 | 6.55 | 6.28 | 5.84 |
| 4 | 5.92 | 6.65 | 6.32 | 6.82 | 6.12 |
| 5 | 6.12 | 6.43 | 7.7 | 7.18 | 7.03 |
| 6 | 6.48 | 7.11 | 7.54 | 7.22 | 6.85 |
| 7 | 7.64 | 6.82 | 6.53 | 7.53 | 5.38 |
| 8 | 6.64 | 5.64 | 6.67 | 7.26 | 6.42 |
| 9 | 6.21 | 6.93 | 7.45 | 6.88 | 6.34 |
| 10 | 6.67 | 5.93 | 7.64 | 7.21 | 7.02 |
| 11 | 6.23 | 6.46 | 7.06 | 6.92 | 6.48 |
| 12 | 7.04 | 6.26 | 6.23 | 7.05 | 5.62 |
| 13 | 6.21 | 5.25 | 6.31 | 7.11 | 6.81 |
| 14 | 6.67 | 5.71 | 7.21 | 6.92 | 5.75 |
| 15 | 6.65 | 6.63 | 7.36 | 7.26 | 5.67 |
| 16 | 6.43 | 6.41 | 7.17 | 6.82 | 6.35 |
| 17 | 7.12 | 6.24 | 6.25 | 6.58 | 6.59 |
| 18 | 6.57 | 6.38 | 6.36 | 7.03 | 5.37 |
| 19 | 6.49 | 5.59 | 7.04 | 5.49 | 6.55 |
| 20 | 6.45 | 6.47 | 6.91 | 6.97 | 5.47 |
| 21 | 6.15 | 6.65 | 5.87 | 7.05 | 6.58 |
| 22 | 6.54 | 5.57 | 6.42 | 6.49 | 6.66 |
| 23 | 6.41 | 7.26 | 7.59 | 5.67 | 6.43 |
| 24 | 6.23 | 6.54 | 6.72 | 6.94 | 5.91 |
| 25 | 6.16 | 7.21 | 5.59 | 5.85 | 6.71 |
| 26 | 5.26 | 5.96 | 7.21 | 6.75 | 6.21 |
| 27 | 6.11 | 6.61 | 6.85 | 6.21 | 6.29 |
| 28 | 6.82 | 5.63 | 7.46 | 5.92 | 6.91 |
| 29 | 6.32 | 6.84 | 6.85 | 5.34 | 6.63 |
| 30 | 7.11 | 7.28 | 7.76 | 6.01 | 7.25 |

**Table S1. Mycelial growth rate of *Pleurotus ostreatus* mushrooms grown on different treatments (raw data).**

Note: T, treatment. CK, control. Note: T, treatment. CK, control. CK: 85% corncob, 12% wheat bran, 3% lime; T1: 85% corncob, 9% wheat bran, 3% BW, 3% lime; T2: 85% corncob, 7% wheat bran, 5% BW, 3% lime; T3: 85% corncob, 5% wheat bran, 7% BW, 3% lime; T4: 85% corncob, 3% wheat bran, 9% BW, 3% lime.

**Table S2. Growth and development of *Pleurotus ostreatus* mushrooms on different substrates (raw data).**

| Treatment | Primordial initiation time (days) | Time interval between flushes (days) |
| --- | --- | --- |
| CK | 42 | 12 |
|  | 44 | 13 |
|  | 44 | 10 |
| T1 | 45 | 13 |
|  | 47 | 12 |
|  | 47 | 12 |
| T2 | 46 | 13 |
|  | 47 | 14 |
|  | 49 | 11 |
| T3 | 49 | 14 |
|  | 46 | 13 |
|  | 46 | 14 |
| T4 | 49 | 14 |
|  | 52 | 13 |
|  | 50 | 13 |

Note: T, treatment. CK, control. CK: 85% corncob, 12% wheat bran, 3% lime; T1: 85% corncob, 9% wheat bran, 3% BW, 3% lime; T2: 85% corncob, 7% wheat bran, 5% BW, 3% lime; T3: 85% corncob, 5% wheat bran, 7% BW, 3% lime; T4: 85% corncob, 3% wheat bran, 9% BW, 3% lime.

**Table S3. Laccase and carboxymethyl cellulase activities at different growth stages of *Pleurotus ostreatus* mushrooms grown on different treatments (raw data).**

|  | Laccase activity (U L^−1^) | | | | | Carboxymethyl cellulase activity (U L^−1^) | | | | | |
| --- | --- | --- | --- | --- | --- | --- | --- | --- | --- | --- | --- |
|  | CK | T1 | T2 | T3 | T4 | CK | T1 | T2 | T3 | T4 |  |
| 10 days after inoculation | 263.57 | 275.21 | 280.56 | 251.35 | 278.65 | 313.38 | 320.49 | 329.01 | 329.57 | 309.41 |  |
|  | 228.93 | 287.86 | 242.59 | 228.89 | 251.78 | 259.18 | 267.81 | 294.41 | 318.28 | 259.62 |  |
|  | 251.29 | 294.68 | 279.12 | 222.73 | 265.43 | 221.47 | 253.58 | 275.47 | 290.03 | 267.15 |  |
| 20 days after inoculation | 213.36 | 229.64 | 248.94 | 265.43 | 245.56 | 247.67 | 275.49 | 342.47 | 298.93 | 247.48 |  |
|  | 235.91 | 252.57 | 217.45 | 243.65 | 223.79 | 196.49 | 246.38 | 314.18 | 283.26 | 229.59 |  |
|  | 215.51 | 261.94 | 217.59 | 251.46 | 256.83 | 268.49 | 242.21 | 259.24 | 247.91 | 197.23 |  |
| 30 days after inoculation | 366.86 | 313.58 | 407.57 | 375.78 | 327.29 | 264.63 | 249.01 | 262.55 | 261.37 | 235.46 |  |
|  | 332.43 | 345.47 | 402.24 | 352.65 | 299.38 | 244.58 | 229.92 | 274.19 | 222.59 | 276.75 |  |
|  | 362.57 | 333.62 | 359.75 | 365.67 | 304.28 | 299.49 | 226.61 | 299.53 | 241.5 | 248.59 |  |
| Primordial initiation period | 159.62 | 143.45 | 191.32 | 152.45 | 138.53 | 128.92 | 169.35 | 199.68 | 167.63 | 187.46 |  |
|  | 196.59 | 184.55 | 223.67 | 172.34 | 141.37 | 163.54 | 143.36 | 218.76 | 196.52 | 167.39 |  |
|  | 161.18 | 172.87 | 205.41 | 188.93 | 159.25 | 112.68 | 132.37 | 231.86 | 168.31 | 133.58 |  |
| Fruiting maturity period | 239.41 | 206.65 | 252.62 | 203.76 | 188.59 | 448.39 | 466.39 | 547.16 | 447.57 | 416.69 |  |
|  | 216.86 | 217.95 | 227.43 | 207.62 | 222.04 | 461.24 | 419.37 | 498.57 | 436.69 | 430.83 |  |
|  | 242.36 | 221.65 | 199.75 | 219.53 | 179.48 | 493.71 | 458.42 | 504.64 | 467.68 | 467.39 |  |

Note: T, treatment. CK, control. CK: 85% corncob, 12% wheat bran, 3% lime; T1: 85% corncob, 9% wheat bran, 3% BW, 3% lime; T2: 85% corncob, 7% wheat bran, 5% BW, 3% lime; T3: 85% corncob, 5% wheat bran, 7% BW, 3% lime; T4: 85% corncob, 3% wheat bran, 9% BW, 3% lime.

**Table S4. Fresh weight of each flush of *Pleurotus ostreatus* mushrooms grown on different substrates (raw data).**

|  | Fresh Weight of the Mushrooms (g bag^−1^) | | |
| --- | --- | --- | --- |
|  | First Flush | Second Flush | Third Flush |
| CK | 676.26 | 476.95 | 257.39 |
|  | 686.41 | 462.51 | 271.29 |
|  | 681.59 | 481.12 | 266.94 |
| T1 | 672.61 | 464.27 | 284.67 |
|  | 689.21 | 479.46 | 269.93 |
|  | 660.74 | 481.77 | 277.89 |
| T2 | 681.35 | 486.59 | 316.12 |
|  | 679.51 | 477.44 | 310.92 |
|  | 673.63 | 482.11 | 329.2 |
| T3 | 640.51 | 465.71 | 285.07 |
|  | 665.96 | 471.56 | 268.75 |
|  | 649.45 | 459.14 | 289.45 |
| T4 | 647.92 | 447.92 | 249.67 |
|  | 639.72 | 469.74 | 232.76 |
|  | 659.57 | 454.11 | 239.02 |

Note: T, treatment. CK, control. CK: 85% corncob, 12% wheat bran, 3% lime; T1: 85% corncob, 9% wheat bran, 3% BW, 3% lime; T2: 85% corncob, 7% wheat bran, 5% BW, 3% lime; T3: 85% corncob, 5% wheat bran, 7% BW, 3% lime; T4: 85% corncob, 3% wheat bran, 9% BW, 3% lime.

**Table S5. Nutritional components of *Pleurotus ostreatus* mushrooms grown on different substrates (raw data).**

| Treatment | Crude Polysaccharide | Crude Protein | Crude Fat | Crude Fiber | Ash |
| --- | --- | --- | --- | --- | --- |
| CK | 4.3 | 18.35 | 0.61 | 4.82 | 7.1 |
|  | 4.26 | 18.26 | 0.67 | 4.91 | 7.15 |
|  | 4.22 | 18.31 | 0.66 | 4.86 | 7.04 |
| T1 | 6.64 | 21.25 | 0.69 | 4.94 | 6.96 |
|  | 6.13 | 21.56 | 0.71 | 5.13 | 6.75 |
|  | 6.27 | 21.62 | 0.75 | 5.05 | 6.71 |
| T2 | 6.62 | 23.31 | 0.63 | 5.22 | 6.91 |
|  | 6.71 | 23.67 | 0.6 | 5.32 | 6.87 |
|  | 6.78 | 23.42 | 0.58 | 5.28 | 6.85 |
| T3 | 8.87 | 20.41 | 0.56 | 6.86 | 6.84 |
|  | 8.82 | 20.37 | 0.52 | 6.78 | 7.02 |
|  | 8.79 | 20.24 | 0.59 | 6.81 | 6.93 |
| T4 | 9.86 | 15.98 | 0.64 | 6.53 | 7.05 |
|  | 9.78 | 16.04 | 0.63 | 6.6 | 6.88 |
|  | 9.87 | 15.66 | 0.66 | 6.66 | 7.1 |

Note: T, treatment. CK, control. CK: 85% corncob, 12% wheat bran, 3% lime; T1: 85% corncob, 9% wheat bran, 3% BW, 3% lime; T2: 85% corncob, 7% wheat bran, 5% BW, 3% lime; T3: 85% corncob, 5% wheat bran, 7% BW, 3% lime; T4: 85% corncob, 3% wheat bran, 9% BW, 3% lime.

**Table S6. Laccase and carboxymethyl cellulase activities at different growth stages of Pleurotus ostreatus mushrooms grown on different treatments (processed data)**

| Treatment | Laccase enzymatic activity（U L^−1^） | | | | | carboxymethyl cellulase activity （U L^−1^） | | | | |
| --- | --- | --- | --- | --- | --- | --- | --- | --- | --- | --- |
|  | 10th days after inoculation | 20th days after inoculation | 30th days after inoculation | Primordial initiation period | Fruiting maturity period | 10th days after inoculation | 20th days after inoculation | 30th days after inoculation | Primordial initiation period | Fruiting maturity period |
| CK | 247.93±17.56bc | 221.59±12.45b | 353.95±18.76b | 172.46±20.91b | 232.88±13.95a | 264.68±46.20a | 237.55±37.05b | 269.57±27.79ab | 135.05±25.98c | 467.78±23.36b |
| T1 | 285.92±9.88a | 248.05±16.62ab | 330.89±16.12bc | 166.96±21.18b | 215.42±7.81ab | 280.63±35.25a | 254.69±18.13ab | 235.18±12.09b | 148.36±18.99bc | 448.06±25.16b |
| T2 | 267.42±21.52a | 227.99±18.14ab | 389.85±26.21a | 206.80±16.22a | 226.60±26.44ab | 299.63±27.15a | 305.30±42.32a | 278.76±18.91a | 216.77±16.18a | 516.79±26.48a |
| T3 | 234.32±15.06b | 253.51±11.03a | 364.70±11.60a | 171.24±18.26b | 210.30±8.22ab | 312.63±20.37a | 276.70±26.13ab | 241.82±19.39ab | 177.49±16.49b | 450.65±15.72b |
| T4 | 265.29±13.44c | 242.06±16.80ab | 310.32±14.90c | 146.38±11.23b | 196.70±22.41b | 278.73±26.84a | 224.77±25.47b | 253.60±21.10ab | 162.81±27.23bc | 438.30±26.16b |

Note: The means ± SD are shown. SD, standard deviation. Letter a, b, c represents the significant difference marker, same lowercase letter indicates no significant differences and different lowercase letters indicate significant differences (α=0.05, ANOVA, LSD test). T, treatment. BW, beeswax waste; CK: 85% corncob, 12% wheat bran, 3% lime; T1: 85% corncob, 9% wheat bran, 3% BW, 3% lime; T2: 85% corncob, 7% wheat bran, 5% BW, 3% lime; T3: 85% corncob, 5% wheat bran, 7% BW, 3% lime; T4: 85% corncob, 3% wheat bran, 9% BW, 3% lime.
